# In vitro models of fetal lung development to enhance research into congenital lung diseases

**DOI:** 10.1007/s00383-021-04864-8

**Published:** 2021-03-31

**Authors:** Soichi Shibuya, Jessica Allen-Hyttinen, Paolo De Coppi, Federica Michielin

**Affiliations:** 1 Stem Cell and Regenerative Medicine Section, Developmental Biology and Cancer Research and Teaching Department, Zayed Centre for Research Into Rare Disease in Children, Great Ormond Street Institute of Child Health, University College London, London, UK; 2 Lungs for Living Research Centre, UCL Respiratory, University College London, London, UK; 3 The Specialist Neonatal and Paediatric Surgery, Great Ormond Street Hospital for Children, London, UK

**Keywords:** Lung development, Branching morphogenesis, Congenital lung disease, Pulmonary hypoplasia

## Abstract

**Purpose:**

This paper aims to build upon previous work to definitively establish in vitro models of murine pseudoglandular stage lung development. These can be easily translated to human fetal lung samples to allow the investigation of lung development in physiologic and pathologic conditions.

**Methods:**

Lungs were harvested from mouse embryos at E12.5 and cultured in three different settings, i.e., whole lung culture, mesenchyme-free epithelium culture, and organoid culture. For the whole lung culture, extracted lungs were embedded in Matrigel and incubated on permeable filters. Separately, distal epithelial tips were isolated by firstly removing mesothelial and mesenchymal cells, and then severing the tips from the airway tubes. These were then cultured either in branch-promoting or self-renewing conditions.

**Results:**

Cultured whole lungs underwent branching morphogenesis similarly to native lungs. Real-time qPCR analysis demonstrated expression of key genes essential for lung bud formation. The culture condition for epithelial tips was optimized by testing different concentrations of FGF10 and CHIR99021 and evaluating branching formation. The epithelial rudiments in self-renewing conditions formed spherical 3D structures with homogeneous Sox9 expression.

**Conclusion:**

We report efficient protocols for ex vivo culture systems of pseudoglandular stage mouse embryonic lungs. These models can be applied to human samples and could be useful to paediatric surgeons to investigate normal lung development, understand the pathogenesis of congenital lung diseases, and explore novel therapeutic strategies.

## Introduction

Impaired lung development can be caused by both genetic and environmental factors. In several conditions, such as congenital pulmonary adenomatoid malformation (CPAM), pulmonary sequestration, congenital diaphragmatic hernia (CDH), and oligohydramnios, the maldevelopment of the lung requires surgical attention. Chronic lung disease of premature babies attributed to lung hypoplasia is also a major problem in neonatal medicine. As congenital respiratory dysfunction threatens the neonate immediately after birth, and current postnatal treatments are suboptimal, novel strategies which aim to rescue impaired lung development prior to birth are desirable. With regard to CPAM and pulmonary sequestration, controversy exists on the efficacy of prophylactic surgical resection. Resection is currently justified by the risk of recurrent infection and theoretically high probability of malignant transformation if left untreated [1, 2]. Given the likelihood that genetic anomalies are involved in their etiology, the answer to the risk of carcinogenesis may lie within the functions of the molecular pathways involved in the pathogenesis of the malformations [3, 4]. From this perspective, the development of in vitro models to understand the mechanisms of lung development will be key to unravel these issues.

Mammalian lung development begins with sprouting of primordial buds from the ventral side of the foregut endoderm. Once the primary lung buds form, they extend into the surrounding mesenchyme and begin the process of branching morphogenesis to finally give rise to the complex airway tree [5]. Lung branching morphogenesis occurs during the pseudoglandular stage (5–17 post-conception weeks in humans, E12–E15 in mice). In mice this process has been well characterized, and branching appears to be highly stereotyped [6]; however, in humans the resulting morphology of the complete airway tree is thought to be more susceptible to variation [7]. Generally, branching is achieved through the elongation and bifurcation of the epithelial bud tips; these consist of Sox9-positive multipotent progenitor cells which give rise to Sox9-negative bronchiolar, and later alveolar descendants [8, 9].

In light of the recent advances, it has become possible to examine biological phenomena at the cellular (and subcellular) level. Investigation of the molecular mechanisms underlying normal lung development, as well as pathogenic conditions associated with the fetal stage, is underway [10]. We additionally have an increased understanding of the key similarities and differences between humans and the mice used as model systems, particularly with regard to developmental mechanisms and the relevant signaling factors involved [9]. Notably, it has been shown that 96% of othologous gene expression between the developing mouse and human lung tip cells are conserved, indicating a high degree of phenotypic similarity between the two [11]. On the other hand, several critical differences have been recognized, such as differential expression of the transcriptional factor SOX2 at the tip of the branching airways. In humans, SOX2 and SOX9 are co-expressed here throughout the pseudoglandular stage; however, Sox2 is consistently absent in mouse epithelial tips [11–13].

Here, we report in vitro models of pseudoglandular stage lung development by using mouse embryonic tissue and a combination of three different approaches, namely whole organ culture, culture of mesenchyme-free epithelium rudiments, and fetal lung organoid culture. These models can be readily applied to human fetal lung samples and will allow investigation of the molecular mechanism of fetal lung development through several avenues.

## Materials and methods

### Whole lung culture

All animal experiments were performed by personnel having UK Home Office Personal Licence (PIL I7ED92582) in line with ethical approval. Wild type CD-1 mice were mated and marked as E0.5 pregnant when they presented a vaginal plug. Pregnant mice were euthanized by cervical dislocation at E12.5, and the embryos were extracted through a laparotomy. Lungs were dissected under a dissecting microscope with extra care to preserve the whole structure. The trachea was divided just below the larynx. After brief washing in PBS, the lung explants were embedded in a 10 μL of 100% Matrigel Growth Factor Reduced (MRF) solution (Corning) dropped on a Transwell® membrane (Corning). After Matrigel drops underwent gelation, 800 μL of DMEM/F12 medium (Thermo Fisher Scientific) supplemented with 1% p/s (Thermo Fisher Scientific) and 0.1% bovine serum albumin, BSA (Sigma-Aldrich) was added to the wells below the membranes. The explants were incubated at 37 °C, 5% CO2, and 21% O2 for up to 72 h. Culture medium was replaced daily.

### Mesenchyme-free mouse lung epithelium rudiments culture

E12.5 mouse lungs were washed in PBS and subsequently treated with 8U/mL Dispase (Thermo Fisher Scientific) for 2 min at room temperature. Mesenchymal tissue was removed with tungsten needles as previously reported [11] and lung epithelium was embedded in MRF drops in 48-well plates. After MRF gelation, 250 μL of DMEM/F12 supplemented with 1% p/s, 0.1% BSA, and 1% Insulin-Transferrin-Selenium (ITS) (Thermo Fisher Scientific) were added. Different combinations of human Recombinant FGF10 (Peprotech) and CHIR99021(Tocris) have been tested as described in Fig. 3.

### Fetal mouse lung organoid culture

Fetal mouse lung organoids were derived as previously reported from bud tips with several modifications [14]. Briefly, mesenchyme-free epithelium rudiments containing at least 3 bud tips were embedded in 20 μL MRF in a 24-well and incubated with advanced DMEM/F12 supplemented with 1% p/s, 1 mM HEPES solution, 1% Glutamax, 1% ITS (all from Thermo Fisher Scientific), Heparin solution (Sigma-Aldrich), 50 ng/mL mouse recombinant Fgf10, 50 ng/mL mouse recombinant Fgf9, 50 ng/mL mouse recombinant Egf (Peprotech), 3 μM CHIR99021, 1 μM p38-MAPK pathway inhibitor BIRB-796, 1 μM A83-01 (all from Tocris), 10 μM Y-27632 Rock-Inhibitor (Cambridge Bioscience). Organoids were expanded by mechanical dissociation and cryopreserved in 50% advanced DMEM/F12, 40% FBS, and 10% DMSO.

### Real-time PCR analysis

Total RNA was isolated with the RNeasy Micro kit (Qiagen), according to manufacturer’s instructions. Reverse transcription to cDNA was performed using the high-capacity cDNA reverse transcription kit (Thermo Fisher Scientific), according to manufacturer’s instructions. Real-time PCR was performed using TaqMan Gene Expression Assay probes (Thermo Fisher Scientific) and Master Mix (Thermo Fisher Scientific) on a Step One Plus Real-Time PCR System (Applied Biosystems). The following TaqMan probes were used: Sox9 (Mm00448840_m1), Sox2 (Mm03053810_s1), and Cdh1 (Mm01247357_m1). Gapdh (Mm99999915_g1) was used as a reference gene. Gapdh was used as an internal control gene to calculate 2^(-ΔCT). Relative gene expression was estimated as 2^(-ΔΔCT) by the Livak method normalizing with the average of 2^(-ΔCT) of E12.5 native lungs.

### Immunofluorescence analysis

Lung explants were released from MRF using cell recovery solution (corning). The specimens were fixed in 4% paraformaldehyde, PFA (Sigma-Aldrich) for 30 min at 4 °C, washed thrice in PBS, and dehydrated and cryoprotected in 30% sucrose overnight. The right and left lungs were separately embedded in Optimal Cutting Temperature compound, OCT (Thermo Fisher Scientific) and stored at -80 °C. Lung organoids were treated with cell recovery solution and fixed in 4% PFA for 15 min at 4 °C. Retrieved organoids were embedded in OCT and frozen at -80 °C. Sections were created at the thickness of 7 and 10 µm for whole lungs and organoids, respectively, using a Cryostat (Leica) and stored at – 20 °C.

After thawing and drying, the sections were permeabilized with 0.5% Triton X-100 in PBS (0.5% PBST) for 10 min at room temperature. PFA-induced background fluorescence was quenched by incubating samples with 50 mM NHCl_4_ for 30 min at room temperature. Then, blocking solution (5% FBS and 0.1% BSA in 0.1% PBST) was added for 1 h at room temperature. The sections were incubated with primary antibodies; goat anti-Human/Mouse E-Cadherin (1:500, AF748, R&D Systems), rabbit anti-Collagen IV (1:500, ab6586, Abcam), and goat anti-Human SOX9 (1:50, AF3075, R&D Systems) diluted in the blocking solution overnight at 4 °C. Secondary antibodies were diluted in blocking solution and included: Donkey anti-Mouse IgG Alexa Fluor® 488 (1:500, A21202, Thermo Fisher Scientific), Donkey anti-Rabbit IgG Alexa Fluor® 568 (1:500, A10042, Thermo Fisher Scientific), Donkey anti-Goat IgG Alexa Fluor® 647 (1:500, A21447: Thermo Fisher Scientific), Hoechst 33342 Solution (62249, Thermo Fisher Scientific), and Alexa Fluor™ 488 Phalloidin (A12379, Thermo Fisher Scientific). After incubation with secondary antibodies for 60 min at room temperature, the slides were washed three times with 0.1% PBST and mounted with Fluoroshield™ with DAPI mounting medium (Sigma-Aldrich). Fluorescence images were captured on a Zeiss LSM 710 confocal microscope (Carl Zeiss) and processed using ImageJ software.

### Statistical analysis

For the epithelial tip culture model, one-way ANOVA test was performed to assess differences in the number of buds at each time point across the different culture conditions. Less than 0.05 of *p* value was regarded as statistically significant. Statistical analyses were performed using R v3.6.3 (The R Foundation for Statistical Computing, http://www.R-project.org).

## Results

### Ex vivo fetal lung culture

**Fig. 1:**
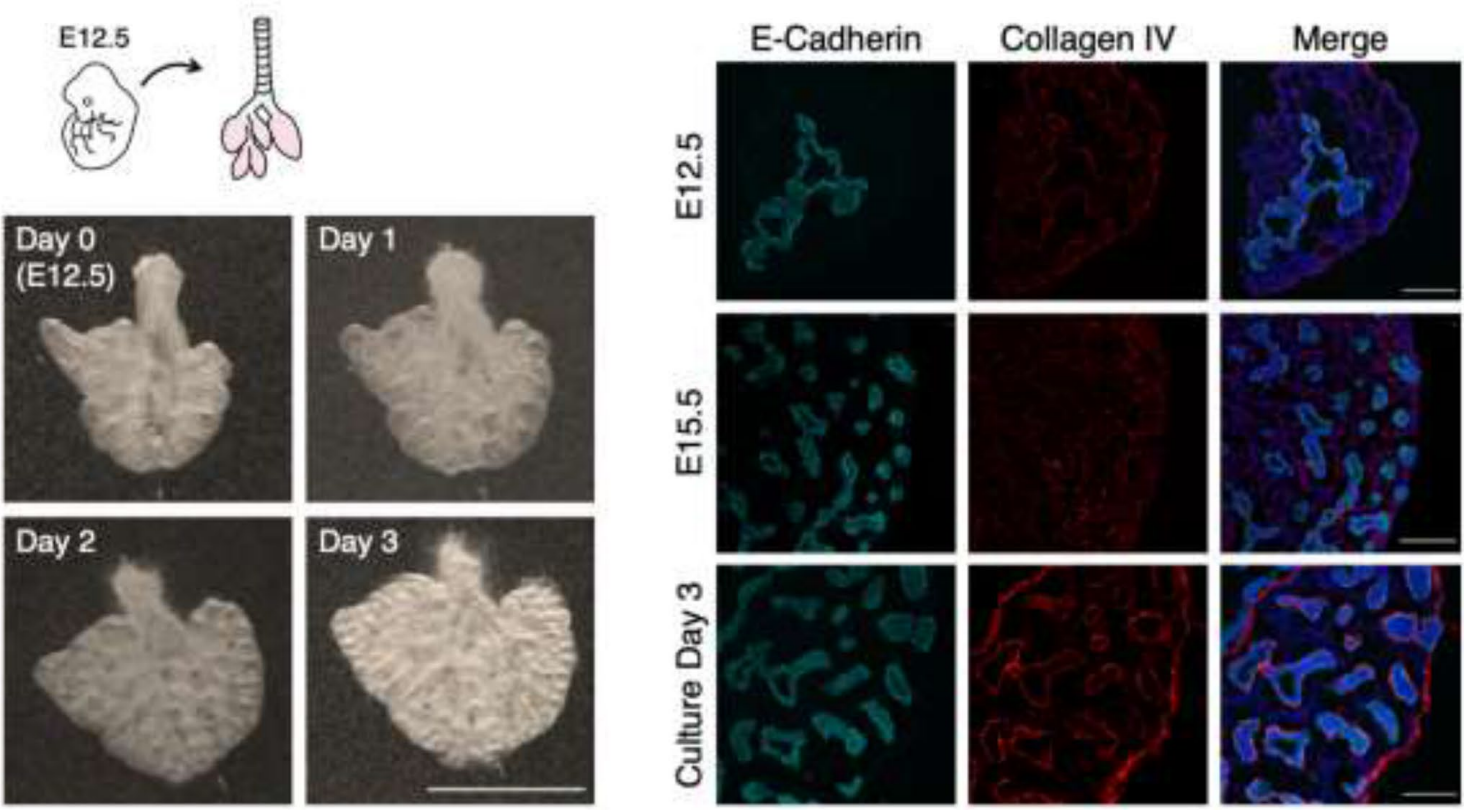
Culture of pseudoglandular stage mouse lung extracted at E12.5 shows branching comparable to native tissue. Left: representative bright field images of a single cultured lung over three days. Growth of the lobes and an increase in the number of buds were observed. Scale bar 100 μm. Right: representative immunofluorescence images of native E12.5 lungs, native E15.5 lungs, and the cultured lungs. The cultured lungs were harvested at E12.5 and cultured for three days, analogous to an E15.5 time point. Scale bar 20 μm

Pseudoglandular stage mouse lung cultures were established to recapitulate branching morphogenesis ex vivo. Branching morphogenesis of lung explants from E12.5 embryos cultured in standard air–liquid interface was assessed by bright field microscopy (Fig. 1). Pictures were captured at 24-hour intervals, demonstrating constant growth of the lungs and generation of new branches. The morphology of the developing airways was compared to E12.5 and E15.5 native lungs, which should correspond to day 0 and day 3 of the cultured lungs, respectively. Although the number of distal buds was decreased compared to E15.5 native tissue, a similar morphology in the cultured sample at day 3 was observed by E-Cadherin and Collagen-4 immunostaining analysis, suggesting a maintained process of branching morphogenesis.

**Fig. 2:**
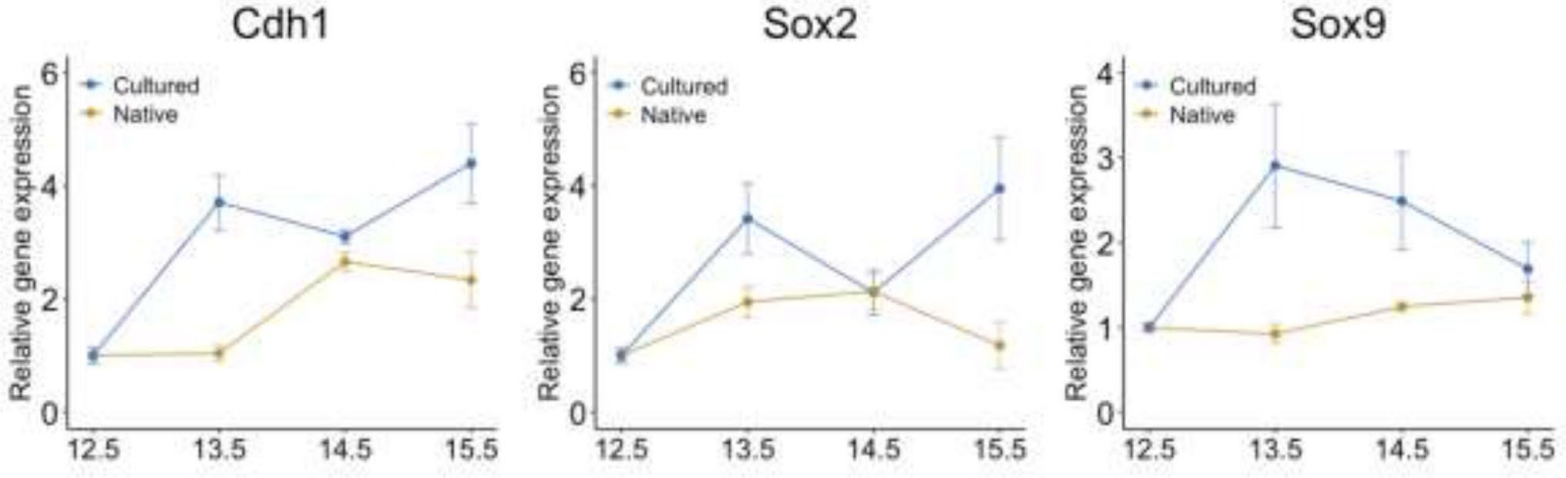
Real-time PCR analysis of Cdh1, Sox2, and Sox9 genes expressed in whole lung culture compared to native tissue at the corresponding developmental stage (E12.5, E13.5, E14.5 and E15.5). Gapdh was used as a reference gene and all values corresponding to cultured and native lungs were normalized to the E12.5 stage. Each time point includes three biological replicates. Data are presented as mean ± SEM

A time course analysis was performed using Real-Time PCR (qPCR) in order to quantify the expressions of key epithelial markers, demonstrating sustained expressions of Cdh1, Sox2, and Sox9 in the cultured specimens over the experimental period (Fig. 2).

### Optimization of culture conditions for ex vivo culture of mesenchyme-free lung epithelium rudiments

The molecular mechanisms which instruct a subgroup of tip cells to undergo symmetry breaking and branching initiation are still not completely understood. However, it has been reported that mesenchyme-free mouse lung epithelium rudiments can spontaneously branch ex vivo if exposed to recombinant Fgf10, showing that pre-patterned mesenchyme is not essential to trigger branching [15, 16]. Hence, the ex vivo culture of fetal lung epithelium represents an ideal model system to investigate the contribution of localized extracellular cues to branching morphogenesis.

**Fig. 3:**
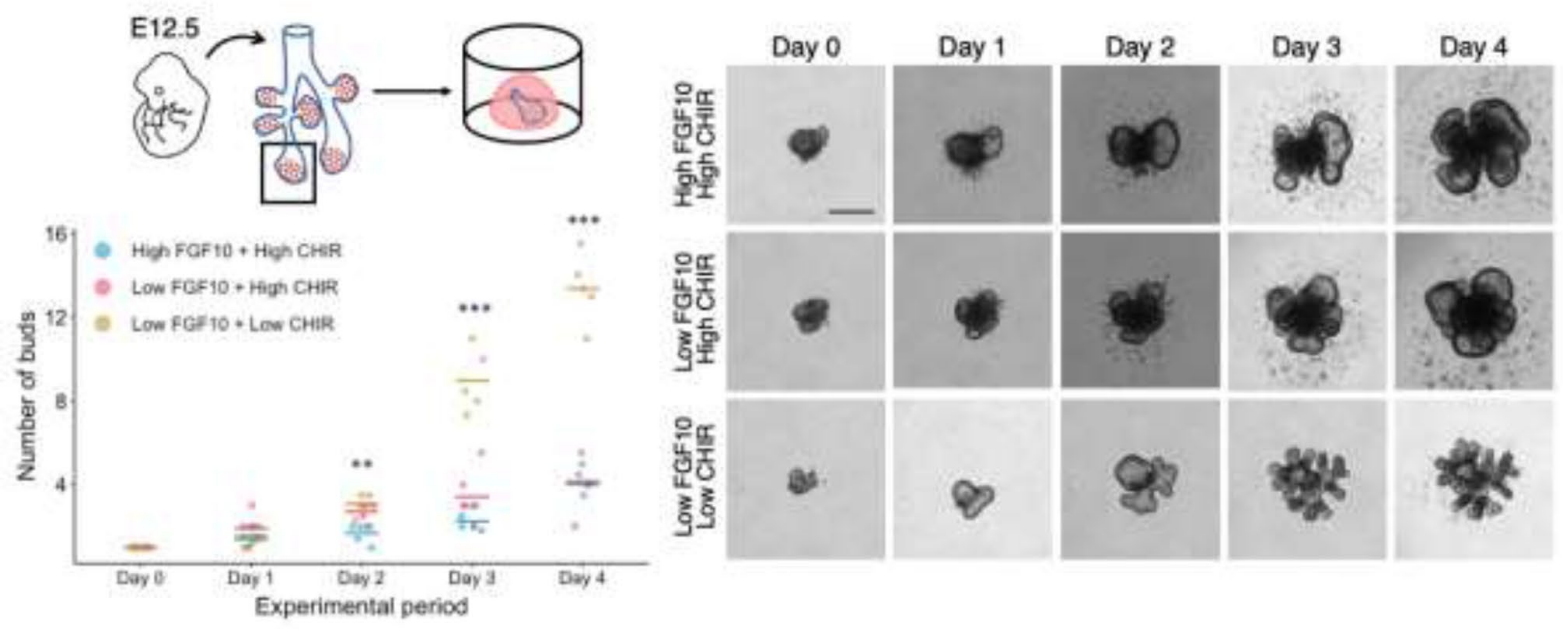
Optimization of branch-inducing culture conditions for epithelium rudiments. Left: quantification of bud tips at 24 h intervals. The number of buds counted on each day during the experimental period was normalized to the initial number of buds plated on day 0. Bars represent the mean of the value and each dot depicts the actual value. Blue: high FGF10 and high CHIR99021, red: low FGF10 and high CHIR99021, yellow: low FGF10 and low CHIR99021, high FGF10: 500 ng/mL, low FGF10: 200 ng/mL, high CHIR99021: 3 μM, low CHIR99021: 1 μM. Each condition includes 3–5 biological replicates. Analysis of covariance; ***p < 0.001; **p < 0.01; *p < 0.05. Right: representative microscopic images of the epithelial tip culture in the different conditions. Bulging of the buds was observed in the high FGF10/high CHIR99021 and low FGF10/high CHIR99021 conditions, whereas the buds in the low FGF10/low CHIR99021 condition displayed productive branching, especially at day 3 and day 4. Scale bar 100 μm

We enzymatically and mechanically removed mesenchyme from E12.5 mouse embryonic lungs and embedded epithelium rudiments in Matrigel drops, to mimic in vivo mechanical stiffness while allowing branching if supplemented with recombinant Fgf10 [17]. Recent studies suggest that the addition of Wnt signaling activators such as the GSK3 inhibitor CHIR992201 enhances branching ex vivo in conjunction with Fgf10 [18, 19]. We sought to further optimize this culture condition by screening different concentration combinations of Fgf10 and CHIR992201, to obtain at least four cycles of epithelial branching starting from Day 0. Specifically, we compared a combination of “high/high” FGF10/CHIR with a “low/high” and a “low/low”. Both the high/high and the high/low culture conditions lead to enlarged tips by day 4, suggesting over-proliferation of Sox9-positive progenitor cells, which prevents formation of new branches (Fig. 3). Conversely, the low/low condition allowed defined branching over 3 days in culture and a significant increase compared to the other two conditions as revealed by quantification of branches normalized to Day 0 (Fig. 3). These results build upon and are consistent with those reported previously [18].

These optimized culture conditions establish an ex vivo model of mesenchyme-free spontaneous lung branching, which may be manipulated to investigate the contribution of specific extracellular cues.

### Testing of an established protocol of self-renewing fetal lung organoid culture

**Fig. 4:**
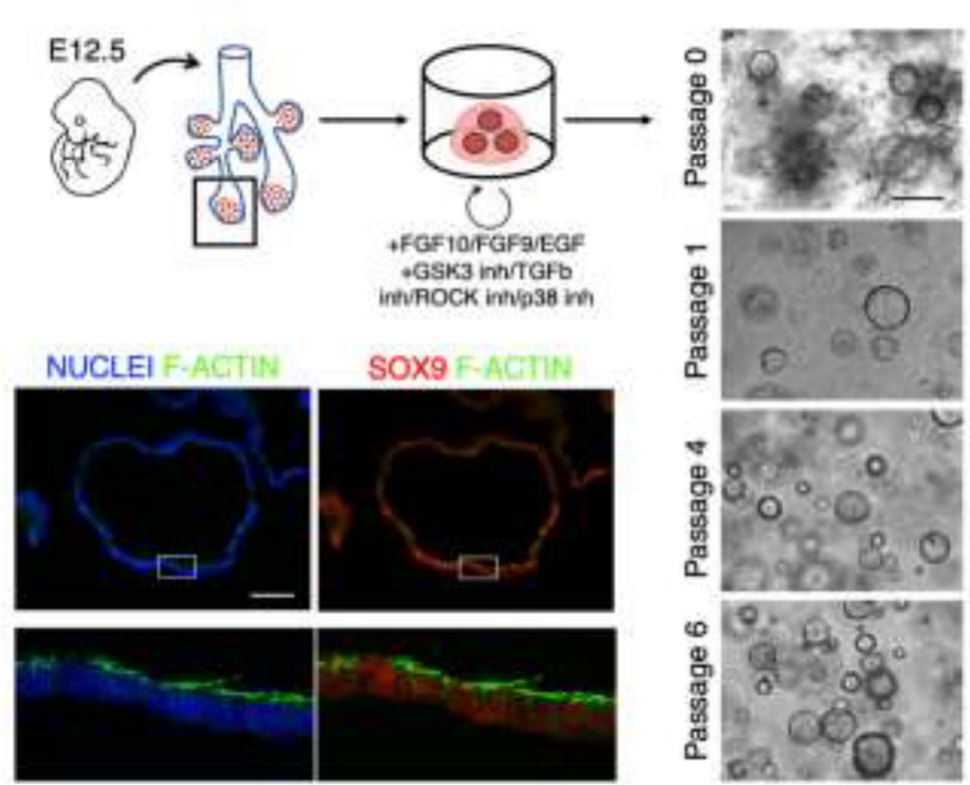
Mouse Organoid culture for self-renewing bud tip cells. Left: representative immunofluorescence images of organoids. In the zoomed pictures (lower row), the top side is the apical (luminal) side. Epithelial cells forming 3D spheroids uniformly expressed positive Sox9 signal. Scale bar 50 μm. Right: representative microscopic images of organoids at different passages. Self-renewing capacity was maintained up to six passages

Fetal lung organoids from bud tip cells allow recapitulation in vitro of the spherical symmetry of a bud tip cell. Taking advantage of an already published study based on the sorting of Sox9-positive cells from E12.5 mouse lung epithelium [14], we tested the potential of mesenchyme-free tips to generate fetal lung organoids without cell sorting (Fig. 4). Immediately after Matrigel embedding residual, mesenchymal cells are still present in the culture. Then, from Passage 1, a homogeneous culture of spherical organoids can be observed that is conserved for at least six passages. The organoids uniformly express Sox9 by immunostaining analysis, along with apical polarization of F-actin, a well-established feature of bud tips in vivo and a key factor in generating the mechanical tension required for branching.

## Discussion

In this work, we present three different models that recapitulate mouse fetal lung development, specifically lung branching morphogenesis, in an ex vivo environment. First, we demonstrated that whole lungs cultured over 3 days maintained expression of key genes related to epithelial branching, and appeared morphologically similar to native tissues. These findings support the value of the ex vivo culture system in the investigation of the early fetal lung development. By virtue of its simplicity, this model has a good potential to study reactions of lungs by applying external factors, such as mechanical pressure, oxidative stress, and nanoparticles loaded with growth factors or microRNAs. Secondly, we recapitulated that mesenchyme-free epithelium rudiments are able to generate new branches if supplemented with an optimized combination of recombinant FGF10 and CHIR99021. This supports the proposed role of mesenchymal cells as a supplier of growth factors and signaling molecules rather than a constructor of the branching morphology [16]. Finally, we tested and slightly modified an established protocol for obtaining fetal lung organoids from the epithelial tips. Sox9-positive organoids represent self-renewing progenitor cells of the airway, which retain both multipotency and proliferative potential, allowing the investigation of differentiation protocols toward bronchiolar or alveolar fate.

Traditionally, research into lung development in the field of pediatric surgery has been based on in vivo studies in animal models [20–22]. However, the utility of animal models is generally limited by the heterogeneity of models and lower cost-effectiveness, making it difficult to validate the observations by repeating the experiments in a consistent manner. Moreover, the difference in gene and protein expression between animals and humans is a hurdle for clinical translation of any discoveries. For example, one of the most widely used animal models of CDH, nitrofen-induced pulmonary hypoplasia, is not replicable in humans, and thus its relevance to human CDH is unclear. Compared to in vivo animal models, ex vivo models provide opportunities to scrutinize phenomena in a more consistent manner and in fine detail at the cellular and subcellular level. The pseudoglandular stage, focused on in this paper, is a crucial period for lung organogenesis, during which disturbance of branching morphogenesis may leave a critical and unalterable deficiency in final lung morphology. In CDH, compression to the lung on the affected side can theoretically happen as early as the mid-pseudoglandular stage, and given that the initial timing of organ herniation varies across patients, whether the lung is compressed during this period may have a large impact on the severity of lung hypoplasia [23]. Therefore, investigating how and to what extent mechanical force affects branching morphogenesis is important for further understanding of the pathogenesis of pulmonary hypoplasia in CDH [24].

Overexpression of FGF10 during the pseudoglandular stage has been demonstrated to induce a formation of cystic lesions resembling CPAM type 1 [25]. In our epithelial tips model, tips treated with high FGF10 or high CHIR99021 exhibited a bulging, cystic morphology without proper separation into new branches. This suggests the necessity of FGF10 regulation for the generation of daughter buds, which is likely to be balanced by Wnt signaling, as indicated by effective branching in the low CHIR99021 condition. This finding is an accordance with a previous report showing cystic malformation caused by deficiency of the Wnt receptor Frizzled 2 in branching airway epithelium [19]. With regard to transcription factors, diffuse expression of SOX9 and SOX2 in the epithelial cells lining the cystic lesion of CPAM type 1 and 2 have been reported ﻿[3, 26]. This suggests disrupted regulation of the respective genes in the cystic lesions. In our whole lung culture, lung explants continued to generate new buds, while epithelial airway genes were also expressed. By manipulating this system, we will be able to obtain quantitative and spatial information of gene and protein expression, providing a new insight in the inception of cyst formation. Understanding these pathways will be the key to addressing the risk of malignancy related to congenital lung malformations.

## Conclusion

Congenital lung malformations are consequences of aberrations in genetic and epigenetic regulation of normal developmental processes. Proper understanding of these mechanisms at the different levels, specifically gene, protein, and tissue levels, is vital for exploring and testing novel therapies, including fetal intervention. We have provided evidence for the utility and reliability of three complementary in vitro models of lung development which have already been adopted in basic biology, but could be particularly useful to paediatric surgeons. These in vitro systems can be harnessed to develop a more comprehensive understanding of the developing fetal lung and will be key to successful translation of basic research to clinical practice.
